# Books

**Published:** 1883-01

**Authors:** 


### Books.

Asthma: Its Pathology and Treatment. By Henry Hyde Salter, M.D., F.R.S. First American from the last English Edition. New York William Wood & Co. 1882. Wood's Library of Standard Medical Authors.

The author of this volume speaks from abundant experience when he says "not only is asthma not an uncommon disease, but it is one of the direst suffering; the horrors of the asthmatic paroxysm far exceed any acute bodily pain; the sense of impending suffocation, the agonizing struggle for the breath of life, are so terrible that they cannot even be witnessed without sharing in the sufferer's distress."

After reviewing the several theories that have, from time to time, been urged as the true pathology of this distressing affection, Dr. Salter gives his own—though by no means peculiar—views on this subject substantially as follows: It is essentially a nervous disease. The paroxysms depend upon spasmodic contraction of bronchial tubes. These phenomena are reflex. The nervous system may be involved in its centres, only in the lungs, or the pneumo-gastric nerve, in both of its branches, may be the seat of the irritation.

The treatment is considered in relation to its application for the relief of the paroxysms and in the intervals of the paroxysms.

For the first purpose, the three drugs with which he—the author—is most familiar and which he recommends, are ipecac, tartar-emetic and tobacco. Of the three, he regards ipecac as the most manageable and it entails the least suffering. Tobacco is the most speedy and effectual. Antimony is liable to produce troublesome nausea and collapse. Chloroform is declared to be one of the most powerful agents in the treatment of the paroxysms that we possess—chloral is not mentioned. Stimulants taken so as to secure their physiological effects are sometimes useful. The fumes of burning nitre paper, though an old remedy, are often useful—the beneficial effects are even striking in many cases. Of the value of iodide of potassium in asthma, our author says that it has not, in his experience, met the expectations based upon the claims of some ardent advocates for it. It is occasionally, however, of great value. He denies that its good effects are due to its influence on the frequently coexisting bronchitis or the gouty or rheumatic condition; for in cases in which these complications are absent the results of its use are as satisfactory as in others in which they are present. He is unable to offer any satisfactory explanation of its occasional undoubted utility.

There is much interesting and valuable matter comprised in this book, prepared by one whose whole soul was evidently thrown into the study and elaboration of his ubject, and the reader cannot fail to be impressed by the earnest and truthful tone of the narrative.

---

Medical Electricity: A Practical Treatise on the Applications of Electricity to Medicine and Surgery. By Roberts Bartholow, A.M., M.D., L L.D. Second Edition Enlarged and Improved. With One Hundred and Nine Illustrations. Philadelphia: Henry C. Lea's Son & Co. 1882. Pp. 286.

Within a year from the time of reviewing the first edition of Professor Bartholow's excellent treatise, we have the pleasure of again commending, in the second edition, the many points of excellence and advantage that impressed us in scanning the first.

The author, in revising the work, has not deprived it of its practical character which so thoroughly adapts it to the requirements of the general practitioner, and which renders it so satisfactory a guide in the study and application of electricity; but he has added about thirty pages to the volume and otherwise increased the amount of matter, besides developing more fully the modern methods of ascertaining and expressing current strength, tension, resistance, etc., and has entered more minutely into the discussion of the polar method, and the action and uses of the magnet.

The work of the publishers is a source of real delight, for it is a genuine pleasure to read a book, apart from its intrinsic merits, that is printed in so unexceptionable a manner. The paper is good, the type very large and clear, and the binding attractive and substantial.

---

THE PHARMACOPŒIA OF THE UNITED STATES OF AMERICA. Sixth Decennial Revision. By Authority of the National Convention for Revising the Pharmacopœia, Held at Washington, A.D., 1880. New York: William Wood & Co. 1882.

This volume of four hundred and eighty-eight pages represents and constitutes the law of this country, pharmaceutically speaking, for the present decade.

It is interesting to note, in the historical introduction, the march toward perfection which this whole subject has made since the formation of the national pharmacepœia in 1820.

In the present edition a number of noteworthy changes have been made. The alphabetical arrangement is complete and is observed throughout the book. The division hitherto existng under the heads "Materia Medica" and "Preparations" has been abolished. The Latin names of alkaloids have been made to terminate in —*ina*, and the corresponding English names in —*ine*. Hence we now have morphina instead of morphia, quinina instead of quinia, but morphine and quinine as heretofore. The so-called neutral principles have received the termination —*inum*, English —*in*, as, for instance, Picrotoxinum, Picrotoxin; santoninum, santonin, etc. Various special alterations have been made in nomenclature, which it is needless to enumerate in detail. Lupulinum and glycerinum take the place of lupulina and glycerina. Sulphidum for Sulphuretum, Manganum for Manganesium; Bromum, Chlorum and Iodum for Brominium, Chlorinium and

Iodinium. It is Chlorate of Potassium, and it is only orthodox to spell assafœtida with one *s*. Latin nouns in —*as* and —*is* are changed to the masculine gender.

A number of obsolete and unused drugs and preparations have been dismissed. The last Pharmacopœia contained 970 titles. In this edition 229 titles have been dropped and 256 new titles have been added. So that we find about one-fourth of the old titles supplanted by the new.

An elixir of orange—as a convenient vehicle for nauseous or unpalatable substances—has been admitted.

The strength of the fluid preparations of opium, excepting paregoric, has been increased, so that sixteen minims of the tincture now represents twenty-four minims of the Pharmacopœia of 1870.

All reference to doses is omitted.

In addition to the subject-matter proper of the volume we find appended a list of reagents. Table of elementary substances. Table of thermometric equivalents. Tables of percentages and specific gravity. Tables of solubility. Saturation table. Lists of articles added to and dismissed from the Pharmacopœia. Lists of changes of officinal and Latin and English titles. Tables showing changes in the strength of preparations. Tables of weights and measures, and a good index.

The work of the publishers accords with the reputation of the house whence the volume emanates, and cannot fail to prove satisfactory.

As a matter of course, the sale of this pharmaceutical code will be immense, as it is essential to every pharmacist; and while it is not absolutely indispensable, it will surely be useful and instructive to every medical practitioner.

We are warned by the publishers that the necessary commentaries will soon be forthcoming. These days he who reads must run—if we may be permitted to paraphrase a scriptural declaration to suit the times in which we live.

---

The Physicians' Visiting List and Diary for 1883. Louisville, Ky.: Geo. H. Deitz & Co.

Messrs. Deitz & Co. have succeeded in presenting to the profession a conveniently arranged pocket record, which contains many useful bits of information, in addition to the usual ruled pages adapted to the various memoranda, which it is important for the active practitioner to have within easy reach. A schedule of fees as a suggestive basis of charges is, among other items, a practical feature of the book.

---

The Medical Record Visiting List, or Physicians' Diary for 1883. New York: William Wood & Co.

The publishers say that in the preparation of this new "visiting list" nothing has been omitted which is believed to be necessary; but useless text, increasing the size of the volume has not been included. It has been sought in every way, they claim, to make it the most concise, the most compact and the most handsome pocket record for physicians' use, and we must say their claims of excellence are well founded. We fail to discover in this publication room for improvement. It is artistic and complete.

